# Exposure to N-Ethyl-N-Nitrosourea in Adult Mice Alters Structural and Functional Integrity of Neurogenic Sites

**DOI:** 10.1371/journal.pone.0029891

**Published:** 2012-01-04

**Authors:** Vivian Capilla-Gonzalez, Sara Gil-Perotin, Antonio Ferragud, Luis Bonet-Ponce, Juan Jose Canales, Jose Manuel Garcia-Verdugo

**Affiliations:** 1 Laboratorio de Neurobiologia Comparada, Instituto Cavanilles de Biodiversidad y Biologia Evolutiva, Universidad de Valencia, Valencia, Spain; 2 Servicio de Medicina Intensiva, Hospital Universitario La Fe, Valencia, Spain; 3 Behavioural Neuroscience, Department of Psychology, University of Canterbury, Christchurch, New Zealand; 4 Laboratorio de Morfologia Celular, Unidad mixta Centro de Investigacion Principe Felipe-UVEG, CIBERNED, Valencia, Spain; University of Otago, New Zealand

## Abstract

**Background:**

Previous studies have shown that prenatal exposure to the mutagen N-ethyl-N-nitrosourea (ENU), a N-nitroso compound (NOC) found in the environment, disrupts developmental neurogenesis and alters memory formation. Previously, we showed that postnatal ENU treatment induced lasting deficits in proliferation of neural progenitors in the subventricular zone (SVZ), the main neurogenic region in the adult mouse brain. The present study is aimed to examine, in mice exposed to ENU, both the structural features of adult neurogenic sites, incorporating the dentate gyrus (DG), and the behavioral performance in tasks sensitive to manipulations of adult neurogenesis.

**Methodology/Principal Findings:**

2-month old mice received 5 doses of ENU and were sacrificed 45 days after treatment. Then, an ultrastructural analysis of the SVZ and DG was performed to determine cellular composition in these regions, confirming a significant alteration. After bromodeoxyuridine injections, an S-phase exogenous marker, the immunohistochemical analysis revealed a deficit in proliferation and a decreased recruitment of newly generated cells in neurogenic areas of ENU-treated animals. Behavioral effects were also detected after ENU-exposure, observing impairment in odor discrimination task (habituation-dishabituation test) and a deficit in spatial memory (Barnes maze performance), two functions primarily related to the SVZ and the DG regions, respectively.

**Conclusions/Significance:**

The results demonstrate that postnatal exposure to ENU produces severe disruption of adult neurogenesis in the SVZ and DG, as well as strong behavioral impairments. These findings highlight the potential risk of environmental NOC-exposure for the development of neural and behavioral deficits.

## Introduction

The neurotoxic potential and carcinogenic effects of N-nitroso compounds (NOCs) are well established [1]. Primary exposure to NOCs is associated with certain diets, tobacco smoke and other environmental sources [2], [3], [4]. In addition to its widespread application in mutagenesis screens in animal models of various human diseases [5], [6], [7], systemic application of NOCs during development (e.g. transplacental administration) has been used in experimental neuro-oncology to induce brain tumors [8], [9]. N-ethyl-N-nitrosourea (ENU) is a chemical of the family of NOC widely regarded as a biological hazard. ENU causes persistent alkylation of DNA bases in the nervous system with subsequent induction of base mis-pairing, resulting in DNA mutations leading to the over-expression of oncogenes and activation of carcinogenesis-related signaling pathways [10], [11], [12]. Prenatal exposure to ENU generates brain tumors with neuropathological features that resemble those of malignant gliomas, and produces apoptotic cell death and changes in cell cycle dynamics of neural progenitors in the subventricular zone (SVZ), suggesting that ENU is neurotoxic to the stem cell population [13], [14]. Interestingly, postnatal exposure does not seem to induce tumors [15], [16], although the toxicity of ENU towards adult neural progenitor cells is maintained when ENU-exposure occurs postnatally. We have recently shown that postnatal exposure to ENU produces disruption in the SVZ and diminishes the proliferative rate of neural stem cells, *in vitro* and *in vivo* [17]. However, it is unclear how widespread are the effects of postnatal exposure to ENU on adult neurogenesis, and the functional implications of such effects.

Neurogenesis occurs in two areas of the adult mammalian brain: the olfactory bulb (OB) and the dentate gyrus (DG) of the hippocampus [18], [19]. New cells in the OB are generated from neural progenitor cells of the subventricular zone (SVZ) [20]. Throughout adult life, cells born in the SVZ migrate a long distance via the rostral migratory stream (RMS) into the OB, where they differentiate into granular and periglomerular interneurons [21]. The SVZ contains at least four different cell types defined by their morphology, ultrastructure and molecular markers: type A/migrating cells, type B/astrocytes, type C/proliferative precursors, and type E/ependymal cells. Type B cells are considered the adult neural stem cells [22]. Type B cells are also present in the DG, where they generate type D/immature neurons, which later mature into new hippocampal granule neurons. Neurons in the DG are born locally in the subgranular zone (SGZ) and migrate a short distance to integrate in the granular cell layer (GCL) [23].

Accumulated evidence supports a role for adult-generated neurons in behavioral and cognitive functions [24]. It has been suggested that the incorporation of adult-born neurons into the OB is required for plasticity and olfactory discrimination [25], [26], [27]. On the other hand, the hippocampus, together with anatomically related structures of the temporal lobe, is essential for various cognitive processes, including declarative memory, spatial memory and contextual learning [28], [29], [30]. Alterations of adult neurogenesis have been associated with cognitive and behavioral deficits, as shown in rodents treated with some drugs [31], which induce cellular and molecular changes in neurogenic sites, or in rodents exposed to fractionated ionizing radiation, which produce selective damage to proliferating progenitors and neuronal precursors [32], [33], [34].

We previously determined the disrupting effects of ENU on the SVZ. The main goal of the present study was to analyze the long-term structural and morphological changes induced by ENU in the SVZ and DG. We also tested the functional consequences at the behavioral level. The data show that postnatal exposure to ENU alters both the structural and functional integrity of adult neurogenic sites.

## Materials and Methods

### Animals

We used forty-eight 2-month-old CD1 male mice (Charles River Laboratory, Barcelona, Spain). All the animals were housed under a 12 h light/dark cycle with food and water available *ad libitum*. All animal procedures were reviewed and approved by the Ethical Committee for Use of Laboratory Animals of the University of Valencia, and followed the European Communities Council (86/609/EEC) guidelines. Animals were anesthetized by an intraperitoneal injection of 2∶1 ketamine/xylazine (5 µl/g of weight) and perfused with 0.9% saline, followed by 2% paraformaldehyde and 2.5% glutaraldehyde (for electron microscopy), or 4% paraformaldehyde (for immunohistochemistry). Heads were removed and postfixed in the same fixative overnight, and brains were dissected.

**Figure 1:**
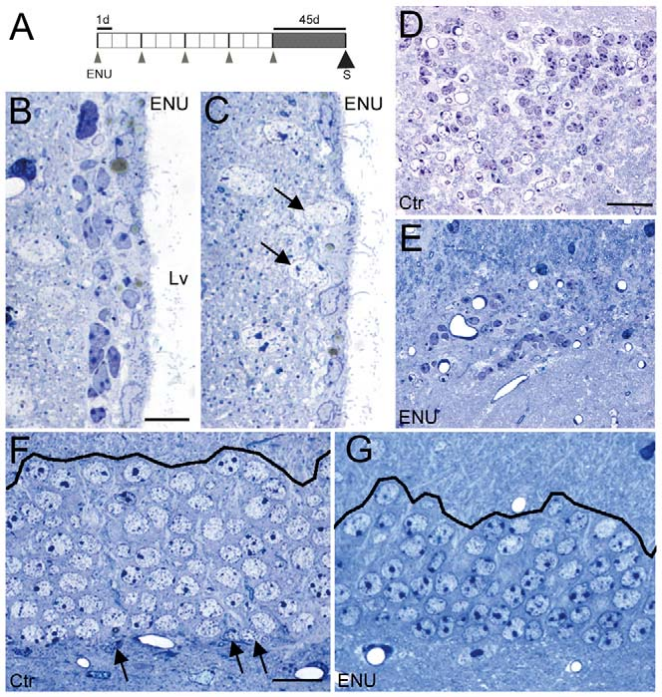
Organization and structure of adult neurogenic niches in ENU-exposed animals. (A) Protocol of ENU administration. We administered 5 injections of ENU separated by 3 days intervals, and mice were sacrificed 45 days after the last dose. (B–C) Semithin sections of the SVZ of ENU animals showed regions with large clumps of dark and light cells (B) alternated with depopulated regions (C) that were reduced to a monolayer of ependymal cells, with neurons located closer to the ventricle lumen (arrows). (D–E) Semithin sections of the RMS of control (D) and ENU animals (E) showed a decrease in the area occupied by migratory chains in treated animals. (F–G) Semithin sections of the DG. (F) Control animals presented frequent neurogenic units in SGZ (arrows). (G) The supragranular region of treated animals showed irregularities, by comparison of control mice. In addition, the experimental group presented scarce neurogenic units in SGZ. Lv: lateral ventricle, S: sacrifice. Scale bar: A 10 µm, C 20 µm, E,G 20 µm.

Mice were exposed to ENU (Sigma Aldrich, St. Louis, MO, USA) by intraperitoneal injections of 100 mg/kg body weight (b.wt.) in a volume of 10 µl/g weight, as described previously [35]. All animals received 5 cumulative doses of ENU (N = 24), one dose every three days, as shown in Figure 1A. Animals were sacrificed 45 days after the last injection. Control animals (N = 24) were injected with the buffer used to dissolve ENU.

### Transmission Electron Microscopy

After post-fixation, brains were washed in 0.1 M phosphate buffer (PB) (pH 7,4), cut into 200 µm sections with a VT 1000 M vibratome (Leica, Wetzlar, Germany) and treated with 2% osmium tetraoxide in 0.1 M PB for 2 h. Then, sections were rinsed, dehydrated through increasing ethanol solutions and stained in 2% uranyl acetate at 70% ethanol. Following dehydration, slices were embedded in araldite (Durcupan, Fluka BioChemika, Ronkokoma, NY, USA).

To study the cellular organization of the neurogenic sites (N_ENU_ = 4, N_CTR_ = 4), we cut serial 1.5 µm semithin sections with a diamond knife and stained them with 1% toluidine blue. Sections were visualized under E200 light microscope (NIKON, Tokyo, Japan). In order to study the changes in RMS size, we determined the area occupied by RMS in coronal semithin sections, at 3 different levels per animal. The analysis was performed with Image Tool software (Evans Technology, Georgia, USA). To identify and quantify cell types, 60–70 nm ultrathin sections were cut with a diamond knife, stained with lead citrate, and examined under a Spirit transmission electron microscope (FEI Tecnai, Hillsboro, OR, USA). To quantify the number of cells within the SVZ, we considered the area comprised in the first 20 µm adjacent to the ventricle lumen (0–1 mm anterior to bregma). In the DG, we analyzed the cells located in the SGZ (1.5–2.5 mm posterior to bregma). The counts were performed on 3 different levels per animal in all cases, and the average was expressed in cells/mm.

### Administration of 5- bromo-2-deoxyuridine

To assess cell proliferation we used the exogenous marker 5- bromo-2-deoxyuridine (BrdU, Sigma Aldrich), which is incorporated into the newly synthesized DNA of replicating cells during the S phase. Animals received a single intraperitoneal injection of BrdU (50 mg/Kg b.wt.) and were sacrificed after 1 h (N_ENU_ = 5, N_CTR_ = 5). To assess cell migration, animals received 4 intraperitoneal injections of BrdU, at 2-hour intervals, and were sacrificed 30 days later (N_ENU_ = 5, N_CTR_ = 5).

### Immunohistochemistry

After post-fixation, brains were washed in 0.1 M PB and cut into 25 µm cryostat sections (Leica, CM 1900) in 6 series for free-floating immunohistochemistry. For each immunoassay, one complete series was used per animal (N_ENU_ = 5, N_CTR_ = 5). Sections were incubated in blocking solution for 1 h at room temperature, followed by overnight incubation at 4°C with primary antibodies (BrdU: 1∶150, cat. no. MO744, Dako, Glostrup, Denmark, USA for single immunostaining; BrdU 1∶200, cat. no. AB6326, Abcam, Cambridge, MA, USA for double immunostaining; Dcx: 1∶200, cat. no. SC-8066, Santa Cruz Biotecnology, Santa Cruz, CA, USA; NeuN: 1∶250, cat. no. MAB377, Millipore, Billerica, MA, USA). Then, sections were washed and incubated with the appropriate secondary antibodies conjugated with either biotin or fluorophores. After the secondary biotinylated antibody, sections were incubated with ABC Elite complex (Vector, Burlingame, CA, USA), treated with diaminobenzidine (DAB, 0.05%; Sigma Aldrich) and later visualized under an Eclipse E200 light microscope. Fluorescence images were taken with a Leica SP2 ABOS confocal microscope.

### Immunohistochemical quantification

The number of positively labeled cells was manually determined by averaging the values from both hemispheres of 3–6 coronal sections per animal (N_ENU_ = 5, N_CTR_ = 5). All cell quantifications were blindly scored under a light microscope.

*Quantification of BrdU-incorporated cells* was expressed as the number of cells per 1 mm along the SVZ or DG for each section. In the case of the OB, results were expressed as the number of cells per 1 mm^2^ for each section. BrdU-NeuN expression was analyzed determining the total number of double positive cells per section and the percentage of BrdU-NeuN positive cells (100*BrdU-NeuN+ cells/total BrdU+ cells).

*Measurement of doublecortin (Dcx) expression* was performed according to previously published protocols for OB and DG. Dcx expression in OB was quantified by measuring integrated optical density and area fraction with ImageJ software (National Institute of Mental Health, Bethesda, MA, USA.) [32]. Because of the heterogeneous nature of the OB, we quantified the staining value in 4 different areas (see Dcx analysis below). Integrated optical density and area fraction were expressed as absolute values because the region of interest was kept constant. In the DG we measured Dcx expression by studying Dcx-negative gaps along the SGZ. A gap was defined as a distance between two Dcx+ cells greater than 3 µm along the SGZ [36], [37]. We quantified the number of gaps (absolute number of gaps and number of gaps/mm), the average length of the gaps, and percentage of surface occupied by gaps.

### Habituation-dishabituation test

A habituation-dishabituation test was used to study the odor discrimination ability of mice 45 days after ENU-exposure (N_ENU_ = 10, N_CTR_ = 10). The test was performed in a Perspex box (21 cm×25 cm×25 cm). The presentations of the odorants were performed with a cotton swab that was impregnated with the relevant odorant and was introduced through a small hole located 8 cm above the floor on one of the side walls. After a 5-min adaptation period, where the swab was presented without odorant during 1-min presentations, 6 consecutive presentations of the Odorant A (habituation phase) were followed by 6 consecutive presentations of the Odorant B (dishabituation phase) of 1 min each [38], [39]. Olive and sunflower oil were used as A and B odorants, respectively. The order of presentation of both odorants was counterbalanced. Tests were recorded by videocamera and analyzed with a nose-tracking software (Viewpoint 2.5, Champagne au Mont D'Or, France), which rendered automatic measures of exploratory activity, measured as time that the mouse's nose is within the boundaries of the swab presentation area (a semicircle of 5 cm in diameter).

### Barnes maze

The Barnes Maze was used to assess spatial reference memory 45 days after ENU-exposure (N_ENU_ = 10, N_CTR_ = 10). The performance in Barnes Maze is known to be highly sensitive to disruption of hippocampal function in rodents and it has been shown to be altered by manipulations of neurogenic function [24], [40]. Moreover, the task is not contaminated by stress, as much as other similar tasks, and no strong aversive stimuli or deprivation is used as reinforcement. The maze design and protocol was based on a previously published protocol [41].

The day before the training phase, a trial was performed in which mice were gently guided to the escape box and left there for 2 min (adaptation period). Animals were trained during 3 days (training phase), performing 4 trials per day with an inter-trial interval of 15 min. The trial ended once the mouse entered into the escape box or after 3 min elapsed. If the mouse did not enter the escape box within 3 min, then the experimenter guided it gently to the hole. The location of the target was kept constant for a given mouse throughout training, but the position was randomized across mice. White noise generated by computer software (Audacity 1.2.6, http://audacity.sourceforge.net/) was used to increase the motivation to escape from the circular platform. The white noise was switched off when the mouse entered into the escape box and mice were left inside the box for 1 min. The maze was cleansed with 30% ethanol and rotated after every trial to prevent bias based on olfactory or proximal intra-maze cues.

Given that mice may sometimes lack motivation and explore the maze after finding the target hole without entering into it, we used the solution proposed in [42] by calculating latency and number of errors made until the target hole was first encountered (primary latency and primary errors respectively). During each trial, both primary and total errors and latency were measured manually by two blind observers. 24 h after the last training day, a reference memory probe test was conducted (testing phase).The escape box was removed and animals were allowed to explore the maze for 90 s. The number of pokes (errors) in each of the holes was measured. Both training trials and test were recorded and analyzed by video tracking software (Viewpoint 2.5, Champagne au Mont D'Or, France).

### Statistical Analysis

Data were analyzed with SPSS 16.0 software (SPSS Inc., Chicago, IL, USA) expressed as means ± SEM. Quantification of BrdU+ cells and behavioral tests, were analyzed with a Student–Newman–Keuls (S-N-K) post-hoc test, after an *F*-test (analysis of variance). Quantification of cell types by transmission electron microscopy were analyzed with a one-tailed Student's *t*-test based on the predicted reduction in cell numbers, as observed in our previous studies [17]. Remaining assays were evaluated with a two-tailed Student's *t*-test after assessing the normal distribution of the data with a Shapiro–Wilk test. The results were considered significant at *p*<0.05.

## Results

### Postnatal exposure to ENU disrupts the organization and structure of neurogenic sites

To study cell organization after ENU-exposure, 5 doses of ENU were injected in adult mice that were allowed 45 days recovery (Figure 1A). In all treated specimens, the SVZ was composed of clusters of light and dark cells (Figure 1B) alternating with depopulated regions, which were reduced to an ependymal layer with neurons being frequently observed in the vicinity of the ventricle (Figure 1C). After ENU-exposure the area of the RMS showed a dramatic reduction of 69% compared to untreated mice (control: 5964±1218 µm^2^; ENU: 1875±347 µm^2^, *p* = 0.0343), but light and dark cells were observed in both groups (Figure 1D–E).

Light microscope images showed that ENU alters the DG, not only in the SGZ, where the number of neurogenic units was reduced, but more clearly in the outer region of the GCL, the supragranular zone, which presented discontinuities compared to control animals (Figure 1F–G).

**Figure 2:**
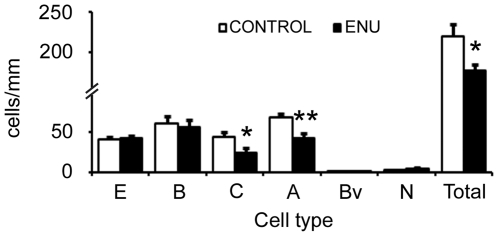
Ultrastructural quantification of cell population in SVZ after ENU-exposure. The bar graph represents the number of cells/mm for the different cell types in the SVZ. The total cell number was reduced significantly by ENU treatment. A reduction was also detected in the number of type C and A cells. However, type B and E cell compartments remained constant. There were not changes in the number of astrocytes contacting with ventricle lumen (Bv) and neurons (N). *p<0.05, **p<0.01.

**Figure 3:**
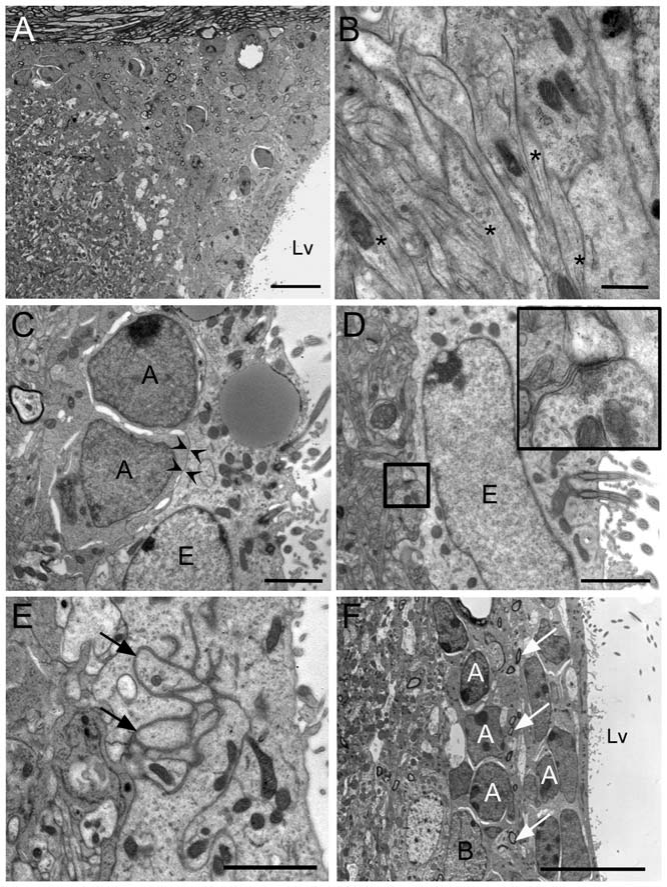
Ultrastructural characterization of the SVZ from ENU-treated animals. The SVZ of treated animals was altered after ENU-exposure. (A) The type A cells located in the dorsal horn of the SVZ in ENU animals were drastically reduced, and substituted by astrocytic expansions. (B) High magnification of the SVZ dorsal horn with expansions rich in intermediate filaments (asterisks) in ENU animals. (C) Ependymal cell and neuroblasts frequently presented direct contact (arrow heads) in SVZ of treated animals. (D) Synaptic contacts located next to ependymal cell in animals exposed to ENU. (E) Large portions of basal membranes were observed between ependymal cells (arrows). (F) Myelinated and unmyelynated axons (arrows) were located between type A cells that composed chains, in ENU animals. Lv: lateral ventricle. Scale bar: A,F 10 µm, B 500 nm, C–E 2 µm.

**Figure 4:**
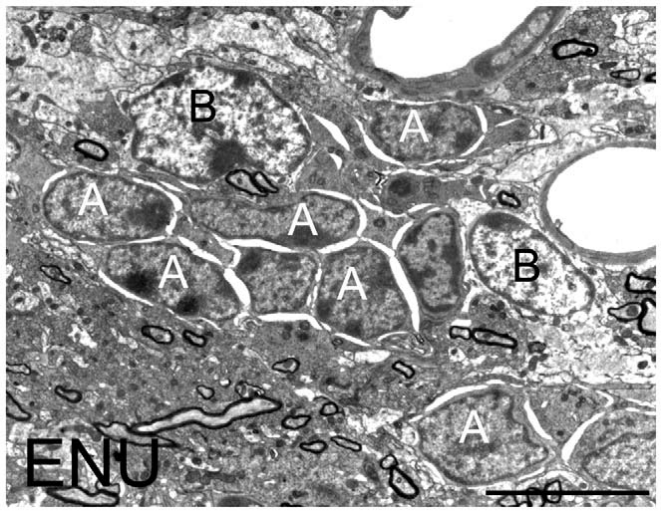
Ultrastructural characterization of the RMS in ENU animals. Detail of a typical neuroblasts chain in the RMS of ENU treated animal, surrounded by astrocytic cells. Scale bar: 5 µm.

Then, we performed an ultrastructural analysis of the SVZ, RMS and DG to identify changes in the organization and cellular composition of these regions. Upon cell quantification, we observed a significant reduction in cell density of the SVZ in treated animals (control: 219±15 cells/mm; ENU: 177±8 cells/mm, *p* = 0.0227). The decrease in cell density was mostly due to a reduction in the number of type C and type A cells (Type C cells: control: 44±6 cells/mm; ENU: 23±7 cells/mm, *p* = 0.02; Type A cells: control: 67±5 cells/mm; ENU: 42±6 cells/mm, *p* = 0.005). The number of ependymal and astrocytic cells remained unchanged (Table 1 and Figure 2A). Most of the neuroblasts located in the dorsal horn were substituted by long and thick expansions of astrocytes in mice exposed to ENU. These expansions were rich in intermediate filaments, resembling the GAP layer described in the SVZ of primates and humans (Figure 3A–B). Although each specific cell type preserved its typical features in terms of the ultrastructure [18], ependymal cells adopted a flattened morphology in regions with cell clusters after ENU-treatment. In addition, ependymal cells frequently contacted directly with type A cells (Figure 3C), and were located next to synaptic contacts (Figure 3D). Deep interdigitations with large portions of basal membranes were intermingled between other ependymal cells (Figure 3E). The analysis depicts that myelinated and unmyelinated axons were located between the ependymal layer and the newly formed migratory chains, a fact that was rarely observed in the control group (Figure 3F). Atypical mitotic bodies were observed in ENU-treated animals, characterized by the presence of large and irregular clusters of dense chromatin. These mitotic cells were B and C cells according to their electrodensity and cell organelles (data not shown). The RMS of ENU-treated mice was reduced in size but showed typical migratory chains consisting of clusters of type A cells surrounded by type B cells. These data revealed no differences in terms of cell morphology by comparison to control subjects (Figure 4).

**Table 1:** Cell quantification in the SVZ after ENU-exposure.

Type cells	cells/mm	
	CONTROL	ENU
**B**	60±9	55±9
**B1**	1,3±0,7	1±0,2
**C**	44±6	23±7*
**A**	67±5	42±6**
**E**	41±3	42±3
**Neuron**	2,9±0,1	4,5±1,1
**Microglia**	1,7±0,6	2,5±0,7
**Pyknotic cell**	0,4±0,3	0,2±0,1
**Not identified**	1,4±0,3	2,7±0,7

*p<0.05,

**p<0.01.

**Figure 5:**
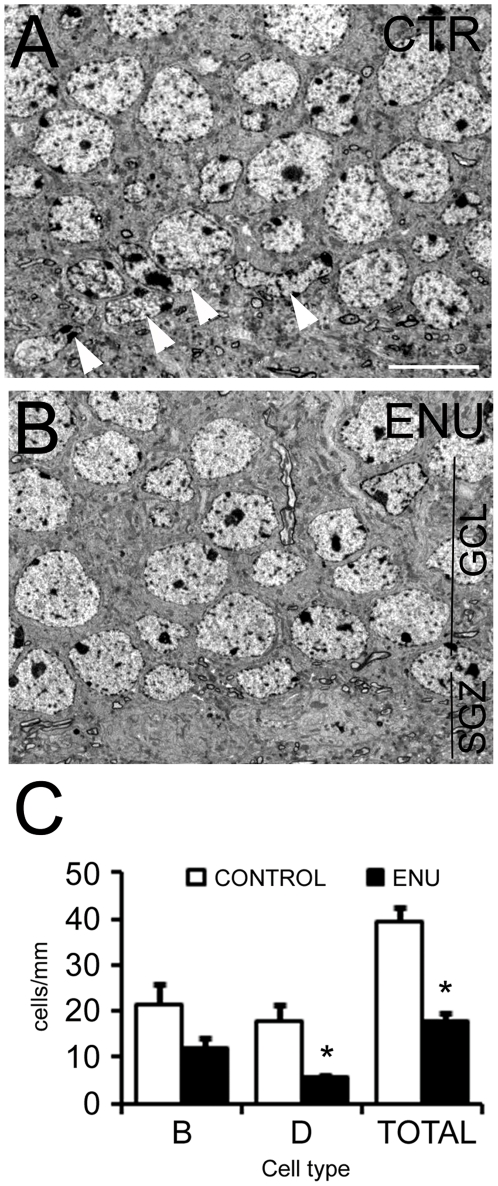
Ultrastructural characterization of the DG. (A–B) Ultrastructural images of the SGZ from DG. (A) The control SGZ showed niches formed by precursor cells (arrow heads). (B) The ENU SGZ did not present the typical niches. (C) Cell quantification of the cell population in SGZ under electron microscopy, measured as cells/mm, resulted in a significantly decrease of total cells, due to a reduction in the number of type D cells. Scale bar: 10 µm *p<0.01.

In order to analyze the cell types in DG we followed previously published criteria [23]. These cells, which constitute the neurogenic niches in the SGZ, did not show relevant ultrastructural changes when compared to the control group. However, we observed a reduction of neurogenic units in SGZ of ENU-treated animals, quantified as total cell number per length unit in this region (control: 39.39±3.06 cells/mm, ENU: 17.91±1.88 cells/mm, *p* = 0.003). In this case the decrease of cell density was due to a reduction in the number of type D cells (control: 18.01±3.42 cells/mm, ENU: 5.71±0.38 cells/mm, *p* = 0.034) (Figure 5A–C).

### Proliferation rate is drastically reduced in SVZ and DG of ENU-treated mice

**Figure 6:**
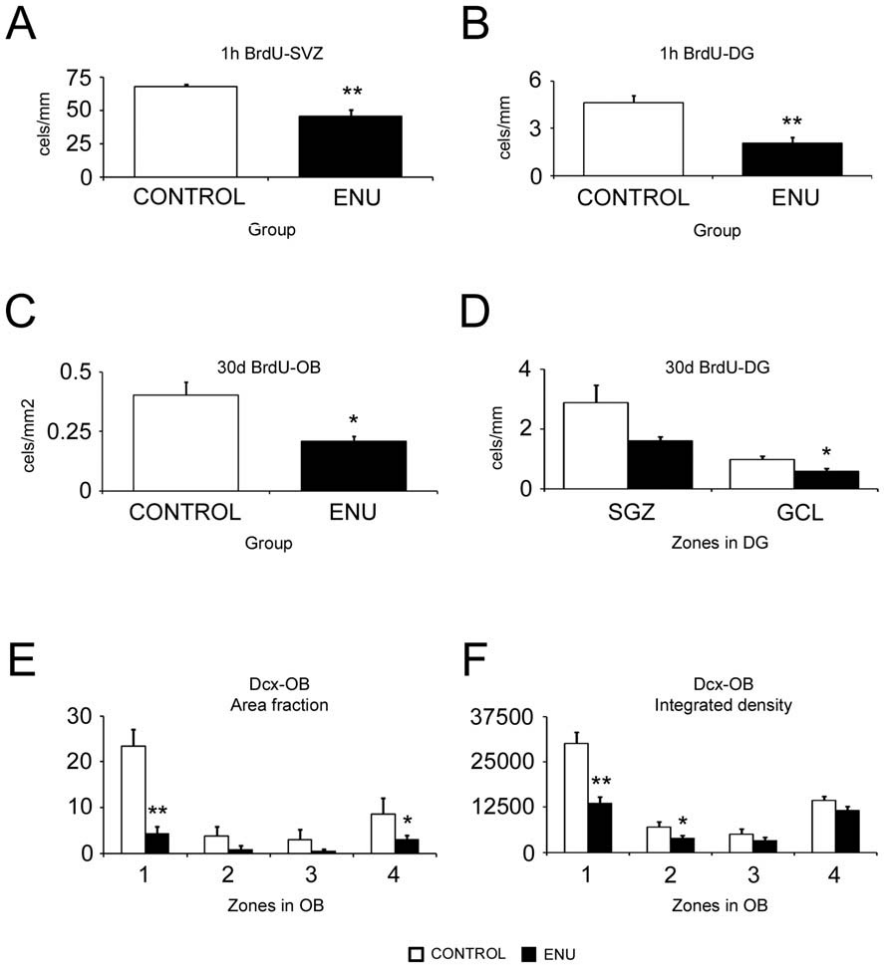
Data analysis of proliferation, migration and early differentiation assays. (A–B) Cells in S-phase 1 hour after BrdU injection. Bar graph depicting the BrdU+ cells/mm shows a significant decrease in the proliferative rate of the SVZ (A) and DG (B) in ENU animals. (C–D) SVZ or SGZ-derived cells 30 days after BrdU injection protocol. Bar graph depicting significant decrease in the numbers of BrdU+ cells in the OB (C) and GCL of the DG (D) in ENU-exposed animals. (E–F) Early differentiation of neurons in OB. Bar graph depicting the area fraction (E) and integrated density (F) of doublecortin (Dcx) positive cells in 4 different regions of OB, showing a reduction in all of them after ENU-treatment. *p<0.05, **p<0.01.

**Figure 7:**
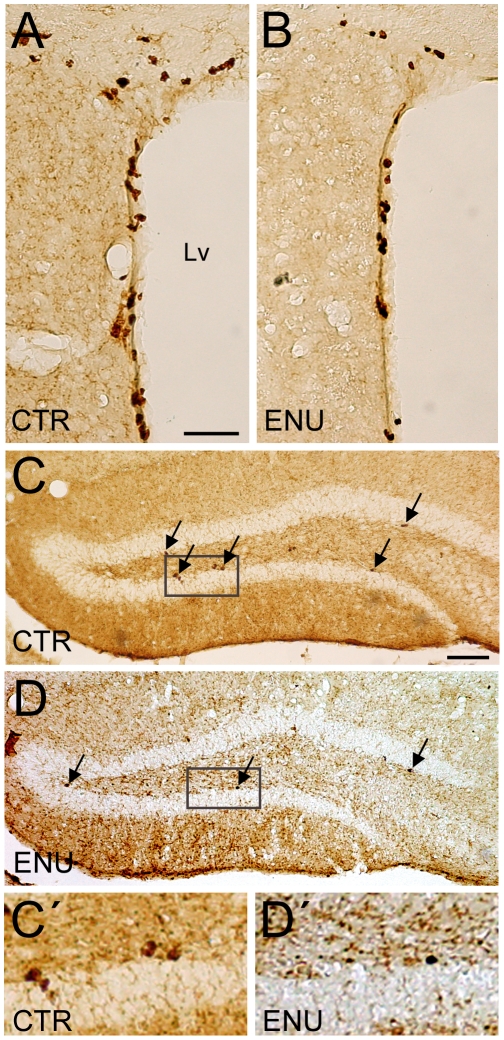
Decrease in proliferation after ENU-exposure as decrease in BrdU immunostaining. Micrograph of the BrdU+ cells immunolabeled with DAB showed a decrease in the proliferation in the SVZ (B) and DG (D) of treated animals, compared with control group (A and C, respectively). Delimited areas in C and D images are enlarged as C′ and D′, respectively, showing a detail of BrdU+ cells in DG. Scale bar: A 50 µm, C 100 µm.

To evaluate the effect of ENU-exposure on proliferation, a single dose of BrdU was given 1 h before sacrifice. Immunohistochemistry was performed to detect BrdU incorporation. Quantitative analysis in SVZ and DG of ENU-treated animals showed a decrease of 33% and 55%, respectively, in the number of BrdU+ cells (SVZ; control: 68.10±1.7 cells/mm, ENU: 45.72±5.1 cells/mm *p* = 0.0089; DG; control: 4.64±0.43 cells/mm, ENU 2.08±10.38 cells/mm, *p* = 0.0021) (Figure 6A–B). In the SVZ of ENU-treated mice, BrdU+ cells formed clusters of 2 to 6 cells homogenously distributed throughout the different SVZ levels (Figure 7A–B). BrdU+ cells in DG after ENU-exposure were observed isolated or forming small clusters of 2 or 3 cells in the SGZ (Figure 7C–D).

### Recruitment of new neurons in neurogenic sites is altered by ENU-exposure

Once we determined that proliferation rate was changed by ENU, we evaluated if ENU-exposure altered the recruitment of neuroblasts in OB and GCL of the DG. Animals were injected with 4 doses of BrdU and sacrified 30 days after treatment. Quantitative analysis in OB showed a significant decrease of BrdU+ cells in mice exposed to ENU (OB: control: 0.4±0.05 cells/mm^2^, ENU: 0.2±0.02 cells/mm^2^, *p* = 0.0223) (Figure 6C). Quantitative analysis in DG also showed a decrease of BrdU+ cells in GCL of DG (GCL: control: 0.99±0.1 cells/mm, ENU: 0.6±0.09 cells/mm, *p* = 0.0123) (Figure 6D). This result shows that the number of new cells recruited in neurogenic areas was reduced after ENU-exposure.

**Figure 8:**
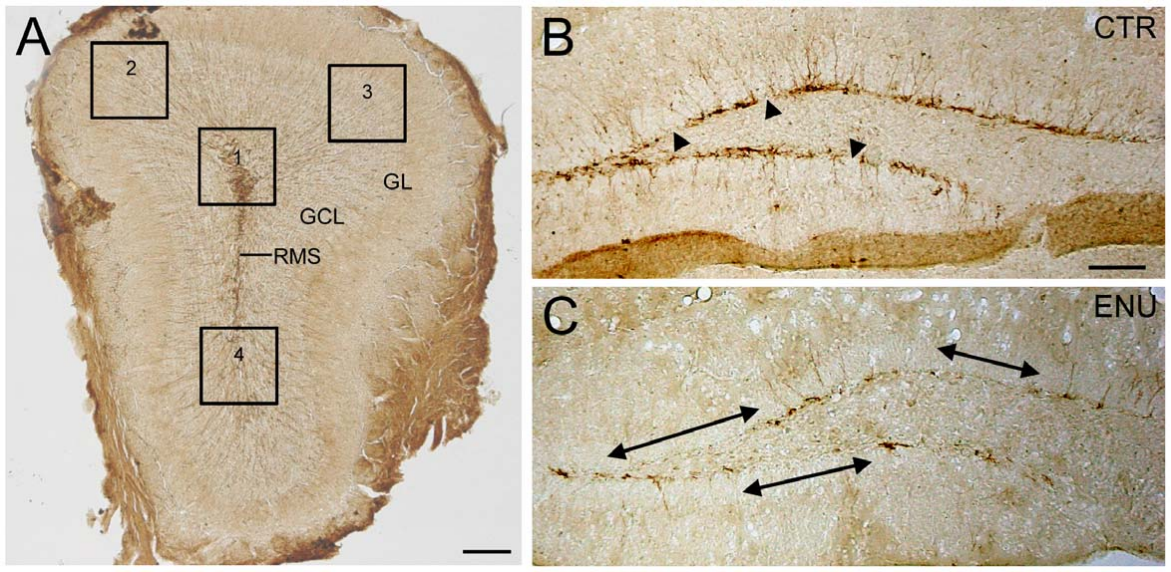
Dcx-expression in OB and DG of ENU-animals. Micrographs of Dcx+ cells immunolabeled with DAB. (A) High magnification of an OB sections from control mouse, where the 4 different regions under study are detailed. Zones 1 and 4 correspond to part of the OB central region, and of the granular layer. Zones 2 and 3 correspond to part of granular and glomerular layers. (B) In control animals Dcx-expression was relatively constant across the SGZ, showing scarce and short gaps (arrowheads). (C) The SGZ in treated animals presented wide and frequent gaps (arrows), compared with the control group. GCL: granular cell layer, GL: glomerular layer, RMS: rostral migratory stream. Scale bar: A 200 µm, B 100 µm.

We also determined a reduction of immature neurons, identified as Dcx+ cells, in OB and DG, supporting our results. To analyze Dcx expression in OB we quantified two parameters: area fraction and integrated density. As OB is a heterogeneous region, we evaluated these parameters in four different zones of the OB (Figure 8A). Zones 1 and 4 correspond to part of the central region of the OB, where the RMS ends, and a portion of the granular layer. Zones 2 and 3 correspond to part of the granular and glomerular layers. We observed that the expression of Dcx was reduced in all regions in ENU animals, being more evident in zone 1 (Figure 6E–F and Table 2). To analyze Dcx expression in DG, we analyzed the gaps devoid of Dcx+ cells along the SGZ. These gaps were scarce and shorter in control animals but frequent and wider in mice exposed to ENU (Figure 8B–C). The average length of gaps was 3 times higher in ENU animals. Furthermore, 58% of the SGZ was devoid of Dcx+ cells following ENU treatment, while the percentage was only 16% in control subjects (see Table 3). This result indicates that ENU-treatment induced a loss of immature neurons in the targeted neurogenic sites.

**Table 2:** Evaluation of Dcx expression in the OB of control and ENU animals.

Parameter	Group	Zone 1	Zone 2	Zone 3	Zone 4
**FracA**	Control	23,5±3,6	3,7±2,2	3,0±2,2	8,7±3,5
	ENU	4,4±1,4**	0,9±0,8	0,6±0,4	3,1±0,9*
**DensIntegr**	Control	30180±3018	6984±1519	5101±1343	14364±1181
	ENU	13588±1792**	3975±780*	3392±802	11614±1074

*p<0.05,

**p<0.01.

**Table 3:** Evaluation of GAPS to Dcx expression in the DG of control and ENU animals.

Group	# GAPS	#GAPS/mm	**µ**m GAPS	Rate (%)
CONTROL	5±0,54	3±0,32	290±51	16±3
ENU	9±0,51*	5±0,28*	987±70*	58±4*

*p<0.01.

**Figure 9:**
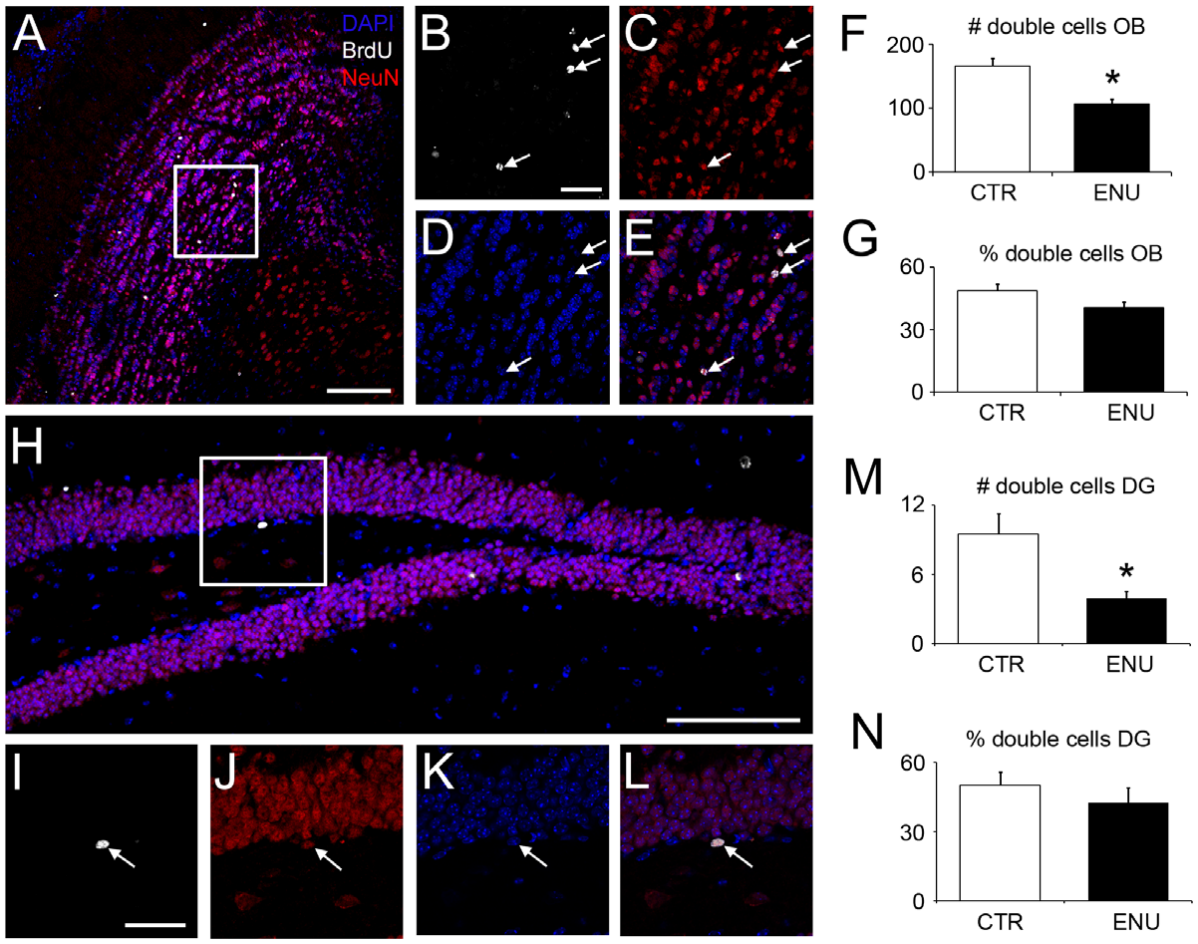
Terminal differentiation in the OB and DG after ENU treatment. (A–E) Images of OB showed NeuN+/BrdU+ cells in granular layer of ENU animals (arrows). (F) Bar graph depicting the number of BrdU-NeuN+ cells in the OB, showing a reduction in ENU animals. (G) The percentage of BrdU-NeuN+ cells in OB did not present differences between groups. (H–L) Images of DG showed NeuN+/BrdU+ cells in ENU animals (arrows). (M) Bar graph depicting the number of BrdU-NeuN+ cells in the DG, showing a reduction in ENU animals. (N) The percentage of BrdU-NeuN+ cells in DG did not present differences between groups. NeuN+ cells (red), BrdU+ cells (white) and DAPI (blue). Scale bar: A and H 100 µm, B and I 30 µm.

To investigate whether the immature neurons (Dcx+ cells) of ENU-exposed animals were capable of terminal differentiation after recruitment in neurogenic areas, we performed double staining with BrdU and NeuN, a marker for mature neurons. ENU-treated mice had fewer BrdU+/NeuN+ cells in the glomerular and granular layers of the OB when compared to untreated mice (control: 166±12 cells, ENU: 107±8 cells, *p* = 0.012). This result was expected given that the number of BrdU+ cells in the OB was also reduced. However, the percentage of BrdU+/NeuN+ cells with respect to the total BrdU+ cells did not show any statistical difference (control: 48.8±2.9%, ENU: 40.6±2.5%, *p* = 0.117) (Figure 9A–G). We observed the same effect in the DG. This region also showed a significant reduction in the number of BrdU+/NeuN+ cells (control: 9.5±1.8 cells, ENU: 4±0.7 cells, *p* = 0.02), but the percentage of cells in which both markers co-localized was not statistically significant (control: 49.9±5.7%, ENU: 42.5±6.6%, *p* = 0.418) (Figure 9H–N). This result indicates that ENU treatment did not affect terminal differentiation.

### ENU-exposure impairs odor discrimination ability in mice

**Figure 10:**
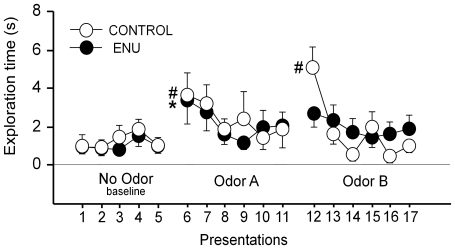
ENU treatment impairs olfactory discrimination. During the habituation-dishabituation test a cotton swab was repeatedly presented to the mice above a target area and changed every minute. Exploration of the target area was examined. After 5 presentations without odorant, the swab was impregnated with an odorant (Odor A), and presented 6 times. Then, another swab was impregnated with a different odorant, (Odor B), which was also presented 6 times. The dotted lines represent the 1 min bins of exploration time of the target area. Notice that both groups similarly detected Odor A (last No Odor presentation Vs first Odor A presentation; * *p*<0.01 for ENU-treated group; # *p*<0.01 for control group), but when Odor B was presented only control animals discriminated the odor difference, responding to the new stimulus (last Odor A presentation Vs first Odor B presentation; # *p*<0.01).

A habituation-dishabituation test was conducted to test the ability of the animals to, first, respond to olfactory cues in general (no Odor vs. Odor A) and, second, to discriminate between two similar odorants (Odor A vs. Odor B). During the adaptation period (5 min exposure to No Odor) both groups spent the same time investigating the target area. The ANOVA showed no effect of either the presentations [F_(4,68)_ = 1.594; *p* = 0.186] or the treatment [F_(1,68)_ = 0.565; *p* = 0.463]. These data indicated that basal exploratory behavior was comparable in both groups before the odorants were introduced. When the first odorant (Odor A) was presented in the habituation phase, both groups of mice were able to detect the new odorant, showing an increased exploration of the target area during the first presentation [F_(5,85)_ = 5.770; *p* = 0.0001]. The last presentation with No Odor was markedly different when compared to the first presentation of Odor A (*p*<0.01, by S–N–K post-hoc test). Thus, both groups of mice were able to detect an olfactory cue (different from no cue). Both groups responded similarly to subsequent presentations of Odor A [F_(5,85)_ = *0.282*, *p* = 0.922]. The time of exploration decreased subsequently during the 6 Odor A presentations (habituation phase), suggesting some degree of habituation of the animals. However, this effect did not reach statistical significance. These data imply that mice treated with ENU did not suffer from impaired exploration at baseline or a general deficit in olfaction. However, we detected differences between groups during the dishabituation phase. Mice exposed to ENU did not show an increase in exploration time, while control mice did, when the second stimulus (Odor B) was presented. The comparison between the 6^th^ presentation of Odor A and the 1^st^ presentation of Odor B for each group, revealed a significant effect of the within factor [F_(1,17)_ = 46.503; *p* = 0.005] and of the interaction with the treatment [F_(1,17)_ = 4.803; *p* = 0.043]. Furthermore, post-hoc comparisons showed that control mice significantly increased the time of exploration (1.61±0.95 s; Vs 5.32±0.97 s, *p*<0.01 by S–N–K post-hoc test) in contrast to ENU-treated mice (1.81±0.71 s; Vs 2.53±0.71). Thus, ENU-treated mice showed an impairment discriminating between different odors (Figure 10).

### ENU-exposure affected spatial memory

To study the functional effects of ENU treatment on spatial reference memory, animals were tested in the Barnes Maze. By comparing the observations made in the first and last training session, we noted that both groups were able to learn the task. All the animals showed a gradual decrease in number of errors (total and primary) and latency (total and primary) to reach the target. The ANOVA indicated significant differences of the within factor in session primary latency [F_(2,36)_ = 10.682; *p*<0.001], total latency: [F_(2,36)_ = 6.818; *p* = 0.003], primary errors: [F_(2,36)_ = 15.271; *p*<0.001] and total errors [F_(2,36)_ = 5.160; *p* = 0.011]. Nevertheless, the main effect of treatment was not significant in any of them. Since no significant effects of the treatment were found for these variables, the effects observed on the training sessions are indicative of comparable learning in both groups (training phase).

**Figure 11:**
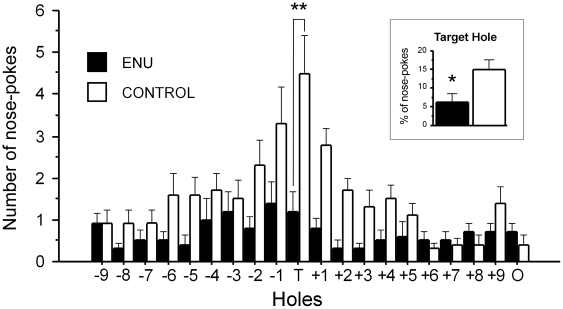
Spatial memory performance in the Barnes Maze is affected by ENU-exposure. ENU-treated animals performed a reduced number of nose pokes in the target hole compared to control animals, thus demonstrating impairment in spatial reference memory. Histograms represent the number of nose pokes in each hole of the Barnes Maze during the test. T: target hole, O: opposite hole to target. The insert graph shows the proportion (expressed as percentage) of nose pokes into the target hole relative to overall nose pokets (*p<0.05, **p<0.01).

However, the probe test performance analysis (number of nose pokes per hole) showed a striking impairment in ENU-treated animals. The analysis showed a significant effect of the treatment [F_(1,342)_ = 9.310; *p* = 0.007] and an interaction effect between treatment and hole [F_(19,342)_ = 15.271; *p*<0.001]. ENU-treated mice showed a reduced number of nose pokes into the target hole in comparison with the control group (control: 4.5±8.975; ENU: 1.2±4.67, *p*<0.01 by S–N–K post-hoc test). Given that ENU- treated animals showed a general decrease in overall nose pokes relative to control values, we performed an additional analysis comparing the proportion of nose pokes into the target hole relative to the overall number of nose pokes. The analysis revealed a significant decrease in the proportion of nose pokes in the target hole in the ENU-treated group [F_(1,17)_ = 6.361; *p* = 0.022] (control: 15.03±2.55 versus ENU: 6.21±2.36), indicating impaired memory in the ENU-treated animals to locate the target (Figure 11). This impairment could not be attributed to reduced exploratory behavior induced by ENU treatment.

## Discussion

This study determined that ENU-exposure disrupts the organization of the adult neurogenic sites after a recovery period, with a decrease in cell proliferation and reduction in the recruitment of new cells in OB and DG. Functional impairments were also detected in odorant discrimination and spatial memory test.

ENU treatment led to an altered cell organization in SVZ and DG neurogenic compartments, and significantly reduced cell density in both regions. There was a selective decrease in the number of fast proliferating precursors (type A and C cells in the SVZ, and type D cells in the DG), while the number of quiescent stem cells (type B cells) remained unchanged. This cell loss resulted in a SVZ with large depopulated regions, similarly to those observed in irradiated [32] and old animals (unpublished results). In DG, the supragranular region displayed irregularities, probably due to a loss of neurogenic units in SGZ, as occurred in GSK3β mutant animals [43]. After ENU-exposure, the ependymal layer showed direct contact with type A cells, and mature neurons were observed in close vicinity of the ventricle. Each specific cell type preserved its typical features, with the exception of the ependymal cells which adopted a flattened morphology in regions with cell clusters. In addition, cell proliferation measured as BrdU incorporation at short survival times, showed a 33% and 55% decrease in the number of BrdU+ cells in SVZ and DG, respectively.

The morphological analysis also revealed a reduction in the size of the RMS, suggesting a decrease in the number of new cells that arrive to the OB. Accordingly, we observed a 50% and 40% decrease in the number of BrdU+ cells in OB and GCL, respectively, when we administered BrdU at long survival times. These phenomena could be explained by two processes: 1) a deficit in migration, or 2) a decrease in progenitors giving rise to neuroblasts. We consider that the second possibility is more feasible, because we demonstrate a significant decrease in the number of neuroblasts that could account for recruitment in the target tissues. Supporting this hypothesis, at least a low percentage of cells are able to migrate correctly towards the OB and the SGZ. However, we cannot rule out the possibility of ENU affecting specifically cell migration. It has been previously demonstrated that ENU induced brain tumors (tissue-specificity) [44], [45], and this effect is influenced by the age of the animal at the time of exposure [16]. However, the relationship between ENU-mutagenesis and the impairment of cell functions, such as cell migration, has not been deeply investigated. Further research would be required to clarify this issue.

Prenatally administered ENU induces the formation of undifferentiated cellular masses, identified as tumors by their histological features [46], [47]. However, postnatal ENU-exposure in our model did not affect terminal differentiation of progenitors, and newly-formed neurons expressed markers of mature neurons, corresponding to NeuN+/BrdU+ cells observed in the target regions. Regarding this finding, our study has a restriction because we did not analyze if new cells are able to incorporate in pre-existing functional circuits. Thus, we do not know if new neurons conclude the maturation process.

In a recent publication, our research group demonstrated that exposure to ENU during adulthood produced an acute decrease in SVZ progenitor cells resulting from increased cell death and reduced proliferative capacity even shortly after ENU-exposure. Four months after treatment, the SVZ was not able to recover the effects generated by the toxin [17]. After a 45-day period of recovery, the changes in SVZ subpopulations could be explained by a likely exhaustion of fast proliferative cells caused by the mutagenic insult and a subsequent halt in proliferation. Although in this study we did not analyze whether there was an increase in cell death, based on our previous data, we hypothesize that cell death is likely to be involved in the process.

There were clear changes in the structural integrity of neurogenic sites following ENU-exposure, but also significant functional deficits. The capacity of discriminating between different odorants and spatial reference memory were impaired in animals exposed to ENU. At the present time, the function of adult neurogenesis remains controversial. Lines of evidence suggest that adult-born neurons in the hippocampus contribute to spatial memory tasks [48]. Some studies reported that newborn hippocampal neurons are required for long-term memory in a variety of spatial tasks using different approaches to reduce neurogenesis, including focused irradiation of the hippocampus [49], genetic manipulations [24], [50], [51] and lentiviral treatment to inhibit WNT signaling [52]. On the other hand, other studies have shown more specific deficits in the flexible use of spatial learning strategies following temozolomide treatment in rats [53]. Some negative results have also been reported determining a mild or no effect in reference spatial memory tasks in Barnes Maze [54], Morris Water Maze [55], [56], and T-Maze tasks [57]. Our data, indicating impairment of Barnes maze performance in ENU-treated mice, are consistent with previous results obtained with irradiated mice using the same task [24], [40]. Furthermore, some studies with aged rodents seem to be consistent with the deficits described in the ENU-treated mice. In these reports a correlation between the extent of spatial memory deficits and reduction in hippocampal neurogenesis has been shown [58], [59]. Nevertheless, our data do not conclude whether ENU exposure induces a general sensorimotor impairment. During the training phase of the Barnes maze, mice exposed to ENU learned the task at the same pace as control mice, exhibiting similar latencies to reach the target hole. Interestingly, they showed impairment during the probe test, which was conducted 24 h after the last training session. The only difference between training and testing phase was the possibility of escape, because the safe escape box is removed from the target hole in the probe test. Therefore, it is likely that the exploratory deficit shown by ENU-treated mice during the probe test is task-dependent. Thus, it may be the consequence of stress associated with removal of negative reinforcement (inescapable stress). Indeed, stress avoidance is the primary motivation to find the safe box in the Barnes maze. Although the Barnes paradigm does not compare directly with paradigms of inescapable stress, the similarity between the primary motivations involved, suggests that this could be a feasible explanation of our results. Moreover, several authors have reported that inescapable stress reduces hippocampal neurogenesis [60], [61], [62], [63] and, conversely, that decreased neurogenesis enhances stress reactivity [64], [65], [66].

The results obtained in the olfactory tasks performed in the current study indicate that mice exposed to ENU do not have a primary olfactory deficit, but rather a deficit in discriminating two different odors. Most evidence accumulated so far do not support a role for newborn olfactory neurons in this type of olfactory task [24], [32], [67], [68]. However, olfactory discrimination has also been shown to be impaired in aged mice with highly reduced neurogenesis [69]. More importantly, our data are consistent with studies in cell adhesion molecule deficient mice, which depict deficit in the migration of olfactory bulb neuron precursors and alterations in odor discrimination, but not in threshold detection of odors [70]. We cannot discard the hypothesis of functional impairments arisen from direct toxic effects of ENU in the nasal neuroepithelium. Nevertheless, the fact that ENU-treated mice were able to respond to Odor A presentations, suggests that this deficit was specific of ENU exposure.

NOCs are formed by the interaction of nitrites and secondary amines [71]. The breakdown of NOCs produces alkylating metabolites, which are causative for the toxic effects on neural cells [14], [72]. Humans are exposed to NOCs in the environment, mainly from preserved food and tobacco [3], [4]. A recent report has suggested that the environment might be a potential risk factor for Alzheimer's disease, especially related to smoking [73]. In the present work adult mice were exposed to NOCs by intraperitoneal injections of ENU. Based on previous work [3], we approximately estimated the NOC amount that a moderate smoking person (10 cigarettes per day) can accumulate during the lifetime. This level of NOC is comparable to a half single dose of ENU administered in our model. However, we have to be cautious when comparing the doses of ENU used in this study with the levels of NOCs found in the environment for different reasons. First, the intraperitoneal administration is not comparable to the environmental exposure (inhalation, transcutaneous, digestion) and this influences the final dose and NOCs bioavailability. Second, to be able to investigate the effect of ENU in our animals, we used an acute exposure to NOCs, far from a chronic lifelong exposure. In this sense our model tended to produce higher toxic effect and affected fewer cells, because the effect on dividing cells occurred at a given time, and not chronically. Finally, the real NOC-exposure depends on multiple factors of each individual (type of diet, smoking, use of certain cosmetics, exposure to rubber products, etc) [2], [3], [4], [74]. Thus, it is complicated to compare our study with the real NOC-exposure. In any case, our results indicate that effects by NOC-exposure should not be ignored.

There are some lines of evidence based on epidemiological data that involve the carcinogenic effects of NOC-exposure on human, but these studies are not conclusive [75], [76]. In addition, the presence of NOCs in the environment is usually minimal, and the human exposure is considered low. However, we consider that the consequences of NOC-exposure are not innocuous. Eventhough NOCs did not induce brain tumours, our data suggest that NOCs could be a risk factor to human health by neurogenesis alteration. This effect on neurogenesis could lead to neurocognitive complications. The relationship between neurogenesis and cognitive functions has been observed in patients under radiation therapy for brain tumors. After treatment, patients frequently experience a progressive cognitive decline [77], [78], which has been related to the effect of irradiation on neural stem/progenitor cells. More recently, it has been demonstrated that not all newly generated neurons in the human SVZ are destined for the OB, but also to the prefrontal cortex [79]. That way, other neurological injuries related to cortical alterations could also result after NOC-exposure.

In conclusion, ENU-exposure had significant effects on SVZ and DG of adult rodents, even after a recovery period. The neurogenic niches were profoundly altered, as a result of decreased proliferating cell populations plus a reduction in both recruitment and early differentiation of new cells. These changes are strongly related to an impairment of the olfactory function and spatial memory. While the extent of the impact of exposure to NOCs on human brain integrity and function remains to be determined, the potential risk justifies further investigation.
